# Anxa2 attenuates osteoblast growth and is associated with hip BMD and osteoporotic fracture in Chinese elderly

**DOI:** 10.1371/journal.pone.0194781

**Published:** 2018-03-23

**Authors:** Xu Zhou, Long-Fei Wu, Wen-Yu Wang, Xin Lu, Zhen-Huan Jiang, Yun-Hong Zhang, Ding-Hua Jiang, Jian-Nong Jiang, Hong-Qing Gao, Shu-Feng Lei, Fei-Yan Deng

**Affiliations:** 1 Center for Genetic Epidemiology and Genomics, School of Public Health, Soochow University, Suzhou, Jiangsu, P. R. China; 2 Jiangsu Key Laboratory of Preventive and Translational Medicine for Geriatric Diseases, School of Public Health, Soochow University, Suzhou, Jiangsu, P. R. China; 3 Municiple People’s Hospital at Yixin, Yixin, Jiangsu, P.R. China; 4 Shishan Street Community Health Service Center at High-tech District, Suzhou, Jiangsu, P.R. China; 5 Department of Orthopaedics, the First Affiliated Hospital of Soochow University, Soochow, Jiangsu, P.R. China; Universite de Liege, BELGIUM

## Abstract

Low bone mineral density (BMD) is a risk factor of osteoporotic fracture (OF). Peripheral blood monocytes (PBM) can differentiate into osteoclasts to resorb bone. It was known that PBM-expressed Anxa2 protein is associated with BMD, and extracellular Anxa2 protein promotes osteoclastogenesis. This study aimed to test 1) whether Anxa2 protein level in PBM differs significantly between subjects with OF and without fracture history (NF); 2) whether Anxa2 level in plasma is associated with BMD; 3) how Anxa2 protein at various concentrations would affect osteoblastic activity *in vitro*. All the study subjects were Chinese Han elderly. Firstly, Anxa2 protein in PBM was identified and quantitated by LC-MS/MS and compared between 45 OF cases and 42 healthy controls. Secondly, plasma Anxa2 protein level was quantitated by ELISA and compared between unrelated subjects with extremely low vs. high hip BMD (0.63±0.10 vs. 1.05±0.10 g/cm^2^, n = 75). Furthermore, *in vitro* functional assay was utilized to test the effects of extracellular Anxa2 protein on osteoblastic growth. We found that Anxa2 protein expression in PBM was significantly up-regulated in OF vs. NF subjects (fold change [FC)] = 1.16, P<0.05). Plasma Anxa2 protein concentration (range: 31.69–227.35ng/ml) was significantly elevated in low vs. high BMD subjects (84.85 vs. 66.15ng/ml, FC = 1.28, P<0.05). Cellular dynamical monitoring demonstrated that the general shape of dose-response relationship is the inverse U-shaped curve. Specifically, lower dose of Anxa2 protein may promote osteoblast growth and the optimal concentration for osteoblastic growth was around 50ng/ml, but even higher concentration could attenuate hFOB1.19 osteoprogenitor cell growth. We concluded that Anxa2 protein could attenuate osteoblast growth and be associated with hip BMD and OF in Chinese elderly.

## Introduction

Osteoporosis (OP) is a major public health problem in the world. It is characterized by low bone mineral density (BMD) and micro-architectural deterioration of bone tissue [1]. Osteoporotic fracture (OF) is the most serious consequence of OP with high disability and mortality. So far, the molecular pathophysiology of OP/OF is still not fully understood yet.

BMD is a widely used classical standard for diagnosing OP [2, 3]. In normal physiological conditions, balanced bone remodeling process is maintained *in vivo* relying on the two counteracting processes of bone formation by osteoblasts and bone resorption by osteoclasts. Imbalanced bone remodeling, for instance, due to excessive osteoclastic bone resorption and/or insufficient osteoblastic bone formation, may result in bone loss and decreased BMD, conferring high risk to OP and OF eventually. Identification of proteins that intermediate bone remodeling would provide novel insights into osteoporosis pathophysiology and benefit for preventive medicine.

Peripheral blood monocytes (PBM) have been demonstrated to be functionally relevant to osteoclastogenesis and OP in humans [4]. The classic PBM (CD14^+^CD16^-^) are precursors of osteoclasts [5]. After migrating from peripheral blood to bone surface, PBM have the potential to differentiate into mature osteoclasts to resorb bone [6, 7]. In addition, PBM produce cytokines important for bone metabolism, such as interleukin-1, transforming growth factor-β, tumor necrosis factor-α, interleukin-6 [8]. After *in vitro* induction, PBM from OP patients presented elevated bone resorption activity in contrast to healthy controls [9]. The above evidences supported PBM as an ideal cell model for OP research.

Our previous two independent proteomics studies consistently highlighted the significance of a PBM-expressed protein, i.e., Anxa2, for OP in humans. Specifically, Anxa2 protein expression level in PBM was found significantly up-regulated in low vs. high BMD subjects in postmenopausal Caucasians [10], as well as in premenopausal Chinese [11]. The above findings indicated that Anxa2 might be an OP risk protein. However, it is still unknown whether PBM-expressed Anxa2 protein level is directly related to OF or not.

Anxa2 is a calcium-dependent phospholipid-binding protein. In bone field, Anxa2 was originally identified as an osteoclasts-secreted protein which could stimulate murine osteoclasts formation [12]. Besides, Anxa2 was found to stimulate monocytes trans-endothelial migration as well [10]. In terms of osteoblastogenesis, functional experiments in Anxa2-deficient MC3T3-E1 mouse osteoblast precursors suggested that Anxa2 could promote osteoprogenitor proliferation and differentiation, hence affect bone formation [13]. In spite of the above evidences from animals and *in vitro* cell cultures, it is unknown whether Anxa2 protein level in plasma is associated with BMD in human populations.

To ascertain the relevance and significance of Anxa2 to OF in humans and for biomarker research and development, the present study was carried out to tentatively address the following two questions. 1) Whether Anxa2 protein expression level in PBM was associated with hip OF in humans? 2) Whether Anxa2 protein level in plasma was associated with hip BMD in humans? In light of the observed negative correlation of plasma Anxa2 with hip BMD, we further pursued *in vitro* cellular functional assay to ascertain whether and how various concentrations of Anxa2 protein could affect osteoblast activity. As a whole, our findings suggest that elevated Anxa2 concentration (>50ng/ml) could attenuate osteoblast growth and be associated with hip BMD and OF in Chinese elderly.

## Materials and methods

### Human subjects

The study subjects were originated from an ongoing Osteoporosis Prevention Project (OPP), a community-based prospective study designed to primarily identify genetic and environmental risk factors of osteoporosis. This study was approved by Institutional Research Ethic Board at the Soochow University. All the study subjects signed informed-consent documents before participating in the study. All subjects were self-identified as Chinese Han individuals.

The study subjects were de-identified from two archived Cohorts, which had been recruited from Shishan Street Community Health Service Center at High-tech District, Suzhou city and Municiple People’s Hospital at Yixin city within Jiangsu province, respectively. Cohort 1 consisted of 363 fracture inpatients (aged 72.1±12.2 years), including 117 males and 246 females. All the patients were recruited during hospital entry day 0–20. Cohort 2 contained 1,860 unrelated elderly (aged 72.0±5.3 years), including 761 males and 1,099 females. Specific information, such as age, height, weight, disease history and medical history, etc., was collected by questionnaire.

Based on the above two archived study Cohorts, two study Samples were generated as follows. Strict exclusion criteria were adopted beforehand to minimize any other known confounding factors influencing bone metabolism or Anxa2 protein expression. Concisely, excluded were subjects with chronic disorders involving vital organs (heart, brain, liver, kidney, and lung), autoimmune-related diseases, metabolic diseases, other skeletal diseases, and hematopoietic diseases. Subjects, who were taking medicine affecting bone metabolism (e.g., glucocorticoid) were excluded as well.

***Sample 1*:** Sample 1 (n = 87) was used to investigate the relationship between PBM-expressed Anxa2 protein and OF risk through a case-control design. Sample 1 contained 45 hip OF patients (>65yrs) from Cohort 1 and 42 healthy subjects without any fracture history (>65yrs; Z-score of hip BMD: -0.05±1.37, mean±SD) from Cohort 2. Approximately 73% OF patients in Sample 1 were recruited during hospital entry day 0–3, and only 2 cases were recruited after day 8. OF is defined as low trauma fractures, i.e., those occurring as a result of falls from standing height or less. In this study, only OF at the site of hip were included as cases in Sample 1. All the hip fractures were confirmed by radiographs or by surgical reports. Cases and controls were well-matched for weight and height. We adopted the analysis of covariance to adjust the covariates age and sex for potential confounding effects. Basic characteristics of Sample 1 were shown in **Table 1**.

**Table 1:** Basic characteristics of the study Sample 1.

	Age (year)	Weight (kg)	Height (cm)
**OF**^a^ (n = 45)	77.8±10.4	58.4±2.5	159.3±1.33
**NF**^b^ (n = 42)	71.2±6.7	58.2±10.5	160.0±1.42
**P value**	<0.001	0.98	0.73

^a^ The OF group include 27 females and 18 males.

^b^ The NF group include 24 females and 18 males.

***Sample 2*:** Sample 2 (n = 75) was utilized to investigate the association between plasma Anxa2 level and hip BMD. Sample 2 was made up of 43 subjects with extremely high hip BMD (hip Z-score: 1.40±0.68) and 32 subjects with extremely low hip BMD (hip Z-score: -1.44±0.45), which were extracted from the top and bottom 8% of the BMD distribution in the elderly population in Cohort 2, respectively. Two subgroups in Sample 2 were matched according to age and height variants. The analysis of covariance was applied to adjust the covariates weight and sex for potential confounding effects. Basic characteristics of Sample 2 were provided in **Table 2**.

**Table 2:** Basic characteristics of the study Sample 2.

	Basic indexes^a^			Bone indexes^a^		
	Age (year)	Weight (kg)	Height (cm)	BMD (g/cm^2^)	P1NP^c^ (ng/ml)	β-CTX^c^ (ng/ml)
**Low BMD**^d^ (n = 32)	71.0±2.6	51.5±7.7	158.1±6.4	0.63±0.10	43.0±19.1	0.23±0.12
**High BMD**^d^ (n = 43)	69.5±2.9	69.1±8.7	160.2±6.5	1.05±0.10	39.7±13.5	0.18±0.11
**P value**^b^	0.2	<0.001	0.56	<0.001	0.39	0.13

^a^ Data are presented as mean ±SD.

^b^ Statistical significance is defined as P<0.05.

^c^ P1NP, procollagen type I amino-terminal propeptide; β-CTX, β-isomerization of the C-terminal telopeptide of type I collagen.

^d^ Low BMD subjects were affected with osteoporosis or osteopenia (T-score: -2.64±0.68). High BMD subject were healthy controls (T- score: 0.47±0.41).

### BMD measurement

Hip BMD (g/cm^2^) was measured by dual-energy X-ray absorptiometry (DXA) (Hologic Inc., Waltham, MA, USA). It is a combined value at three regions including femoral neck, trochanter, and interchochanter. A control phantom consisting of calcium hydroxyapatite embedded in a cube of thermoplastic resin was scanned for daily calibration, and all DXA measurements were performed on the same DXA machine by certified operators. The precision of BMD, expressed as the root-mean-square percent coefficient of variation (RMS-CV), was evaluated by testing thirty volunteers for three times. The RMS-CV for hip BMD was 2.49%.

### PBM isolation for Sample 1

For Sample 1, approximately 10 milliliter peripheral blood was drawn from each subject by certificated phlebotomist, and during day 0–20 for hip OF inpatients. Sodium citrate was used as anti-coagulant. The fresh blood samples were immediately processed for PBM isolation, which was completed on the same day with phlebotomy. Specifically, with the density gradient centrifugation liquid Histopaque-1077 (Sigma,H1077-1), peripheral blood mononuclear cells (PBMC) were firstly isolated from whole blood, then CD14^+^ PBM cells were directly labeled with anti-CD14 magnetic MicroBeads (Miltenyi, Cat No.130-050-201). The mixture of microbeads and CD14^+^ cells was positively selected by retaining within the MACS Column (Miltenyi, Cat No. 130-042-201) in the magnetic field when cell suspension was loaded onto the column, while the unlabeled mononuclear cells passed through the separator. After removing the column from the magnetic field, the magnetically retained CD14^+^ cells were eluted from the column and pelleted through centrifuge.

### PBM-expressed Anxa2 protein quantification by LC-MS/MS for Sample 1

The Anxa2 protein expression data was extracted from an ongoing proteome-wide quantitative proteomics study on OF. For PBM total proteins extraction, 300ul SDT (4% SDS, 1.0mM DTT, 100mM Tris-HCl, PH = 7.6) buffer was added to spall the cell pellets, then the lysate was boiled in water bath for 15 minutes. After high-speed centrifuging for 40 minutes at 14000×g, the supernatant was collected, and the protein concentration was measured with BCA Protein Assay kit (Beyotime, Cat No. P0012S).

During protein sample preparation, three case or control samples were pooled together. Consequently, 15 pooled OF and 14 pooled NF samples were generated. Each pooled total protein sample (200μg) was subject to routine tryptic digestion. The resultant digest concentrations were measured by NanoDrop. Approximately 2.0μg protein digest was subject to separation by Easy-nLC1000 liquid chromatograph (Thermo) and detection by Q-exactive mass spectrometer (Thermo). The raw proteomic data was processed by the softwares Maxquant and Perseus to generate proteome-wide protein expression profile. Specifically, the protein expression level was exported as Label-Free Quantification intensity (LFQ) [14, 15]. Based on the proteome datasets, Anxa2 protein expression data was extracted for statistical analyses between OF and NF groups.

### Plasma Anxa2 and bone metabolic biomarker (P1NP and β-CTX) qualification by ELISA for Sample 2

Fasting blood samples were collected from all subjects in Cohort 2 in the early morning. Plasma was separated on the same day (500×g,10min) and stored frozen at -80°C. The de-identified samples were picked out ahead of time and warmed to room temperature before assayed. The raw plasma samples from the Study Sample 2 were measured in a batch without dilution by using ELISA kit (Neobiolab, Cat No.HA0601) according to the manufacturer’s instructions. The sensitivity of Anxa2 ELISA assay is 1.0ng/ml. No significant cross-reactivity or interference between Anxa2 and any homologous proteins assayed has been observed. The intra-assay and inter-assay CV of the kit were 5.9% and 6.9%, respectively.

In addition, the bone anabolic biomarker, i.e., procollagen type I amino-terminal propeptide (P1NP), and the bone catabolic biomarker in plasma, i.e., β-isomerization of the C-terminal telopeptide of type I collagen (β-CTX) were also quantitated by respective ELISA kits (FaikangBiotec, Cat. No.FC-007 and FC-008; Guangzhou, China).

### Cell culture

Human fetal osteoblastic cell line (hFOB1.19) was obtained from the Institute of Cell Bank/Institutes for Biological Sciences (Shanghai, China, http://www.cellbank.org.cn). The hFOB, serving as osteoprogenitors, has the ability to differentiate into mature osteoblasts under a restrictive condition. For *in vitro* cell growth assay, hFOB cells were maintained at 33.5°C in complete medium consisting of 1:1 DMEM/Ham’s F-12 medium (Life technologies, New York, USA) without phenol red supplemented with 10% fetal bovine serum, 0.3mg/ml G418 (Roche), and 1% penicillin/streptomycin (Invitrogen) in an atmosphere of 5% CO_2_.

### Cell growth assay

The hFOB cells were seeded at approximately 7×10^3^ cells/well in 96-well plates and allowed to adhere for 4–5 hours at 33.5°C in complete medium before treatment. The recombinant protein of human Annexin A2 (Origene, Catalogue No.TP305081) was then added to the medium generating various concentrations (0, 25, 50, 100, 200ng/ml). The experiment was conducted in duplicate for each concentration. According to the set time interval, hFOB growth process was monitored for 36 hours and recorded as specific cell indexes (CI) in real time by the RTCA S16 instrument (Model:1×16, Serial No: 58-1-1509-1086-4, China) following the manufacturer’s instructions. The dynamic growth trends during 36 hours were exported as time-dependent graph.

### Statistical analysis

All statistical analyses were performed using GraphPad Prism 5 software. The majority of continuous variables were described as mean±SD. Differences in Anxa2 protein level between groups in PBM (OF vs. NF) in Sample 1 and in plasma (high vs. low BMD) in Sample 2 were analyzed by using two-sided Student’s t test or Unpaired t test with Welch’s correction. The same methods were also used to test the differences in sex-stratified Sample 1 and Sample 2, respectively. A scatter plot was drawn to observe the possible correlation between PBM-expressed Anxa2 protein level and the hospital entry day. The analysis of variance was applied to test the difference in cell growth state among multiple concentrations. The analysis of covariance was applied to adjust for the confounding effects of significant covariates in Sample 1 and Sample 2. Pearson correlation analysis was performed to test the association between plasma Anxa2 and hip BMD in Sample 2. P value less than 0.05 was set as statistically significant.

## Results

### Significant elevated Anxa2 protein expression in PBM in OF vs. NF subjects

**Fig 1:**
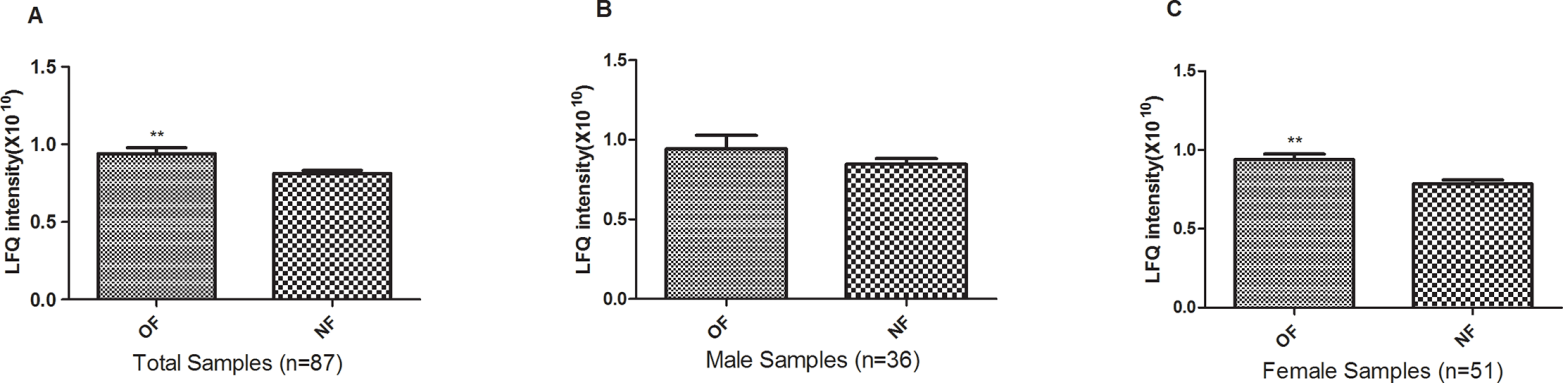
Up-regulation of PBM-expressed Anxa2 protein in OF versus NF subjects. Presented are Anxa2 protein abundances (Label-Free Quantification [LFQ] intensity) in PBM in Sample 1. The data is described as mean±SD. **: P<0.01, as compared with NF subjects.

The scatter plot (**S1 Fig**) indicated that there was no significant correlation between Anxa2 protein expression level in PBM and hospital entry day (P = 0.46). Two-sided student’s t-test showed that the Anxa2 protein expression level in PBM was significantly elevated in the OF group compared to the NF group (FC = 1.16, P = 0.007, **Fig 1**). The difference remained significant even after adjusting the covariate sex (P = 0.014). Gender-stratified analyses revealed that the difference remained significant in the elderly females (n = 51, P<0.01). Although the difference does not reach significance level in the elderly males, the same trend of elevated expression in the OF group was also observed. Considering the effect of covariate age on Anxa2 expression in PBM, we adopted the analysis of covariance to adjust the confounding effects. The difference remained significant in total Sample 1(P = 0.027), as well as in the elderly females (P = 0.037).

### Significant negative correlation of plasma Anxa2 with hip BMD in Sample 2

**Fig 2:**
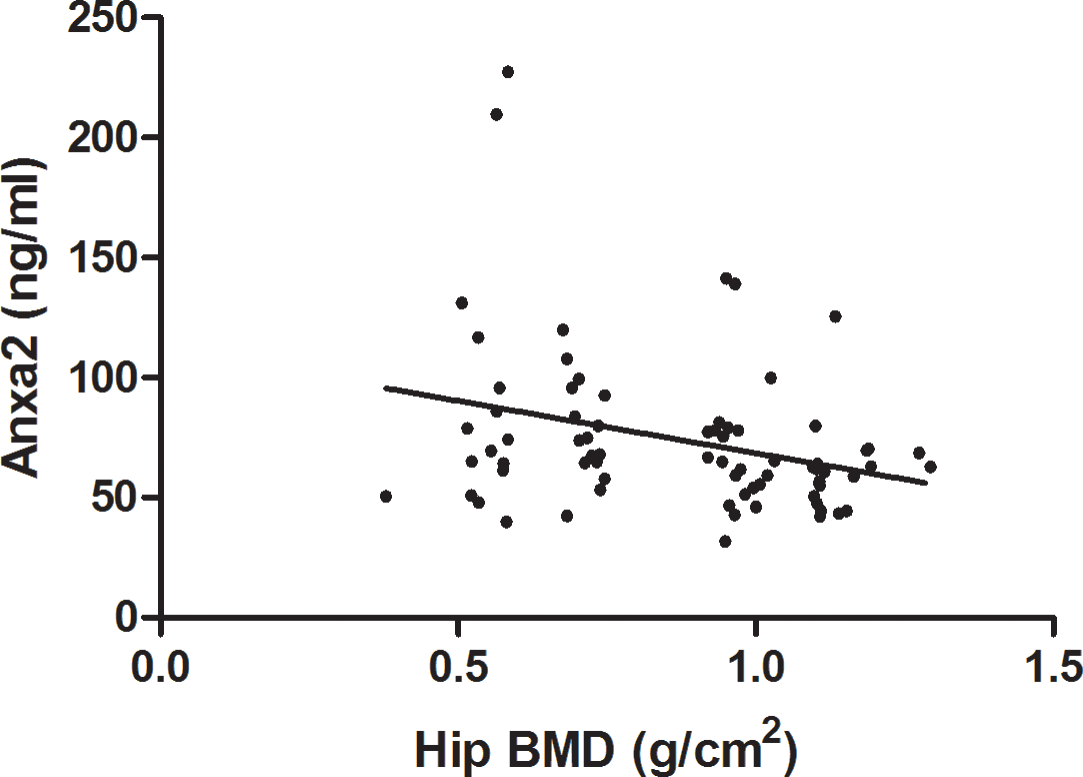
Plasma Anxa2 protein level was negatively correlated with hip BMD in Sample 2. The correlation between Anxa2 protein level in plasma and hip BMD was analyzed with Pearson correlation analysis (r = -0.302, P = 0.0084).

**Fig 3:**
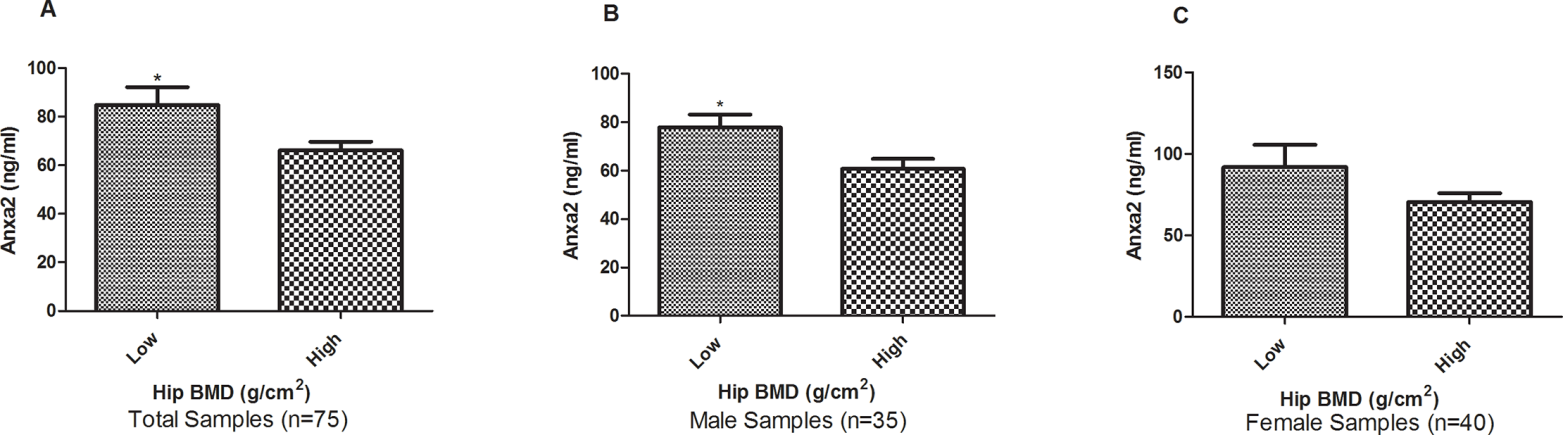
Up-regulation of plasma Anxa2 protein in low versus high BMD subjects. Presented are Anxa2 protein abundances in plasma in Sample 2. The data is described as mean±SD. *: P<0.05, as compared with high BMD subjects.

The standard curve for plasma Anxa2 ELISA assay was generated by following the product instruction using specific four-parameter logistic fitting model (r = 0.99, **S2 Fig**). ELISA assay showed that the plasma Anxa2 protein concentration ranged from 31.69 to 227.35ng/ml in the Chinese elderly in the Sample 2. Pearson correlation analysis showed that Anxa2 protein level in plasma was negatively and significantly correlated with hip BMD in Sample 2 (r = -0.30, P = 0.008, **Fig 2**). The plasma Anxa2 level was significantly elevated in subjects with extremely low BMD compared to subjects with extremely high BMD subjects (84.85 vs. 66.15ng/ml, P = 0.027, **Fig 3**). The elevation remained significant even after adjusting the covariate sex and weight (P = 0.012).

Gender-stratified analyses revealed that the difference remained significant in the elderly males (N = 35, 77.87 vs. 60.68ng/ml, P = 0.014). Although the difference does not reach significance level in the elderly females, the same trend of elevated expression was observed in the extremely low BMD group (N = 40, 91.82 vs. 70.48ng/ml, P = 0.17). In contrast, no significant difference in plasma P1NP and β-CTX levels were observed between the low vs. high BMD subjects (**Table 2**).

### Dual effects of Anxa2 protein on osteoblastic growth

Dynamic monitoring of cell growth for 36 hours demonstrated that the effect of recombinant protein of human Anxa2 on hFOB growth is concentration-dependent (**S3 Fig**). Compared to blank control without Anxa2 supplement, Anxa2 at various concentrations (25-200ng/ml) generally exerted a promotive effect on osteoblastic growth, and the optimal concentration was around 50ng/ml (**S3 Fig**).

**Fig 4:**
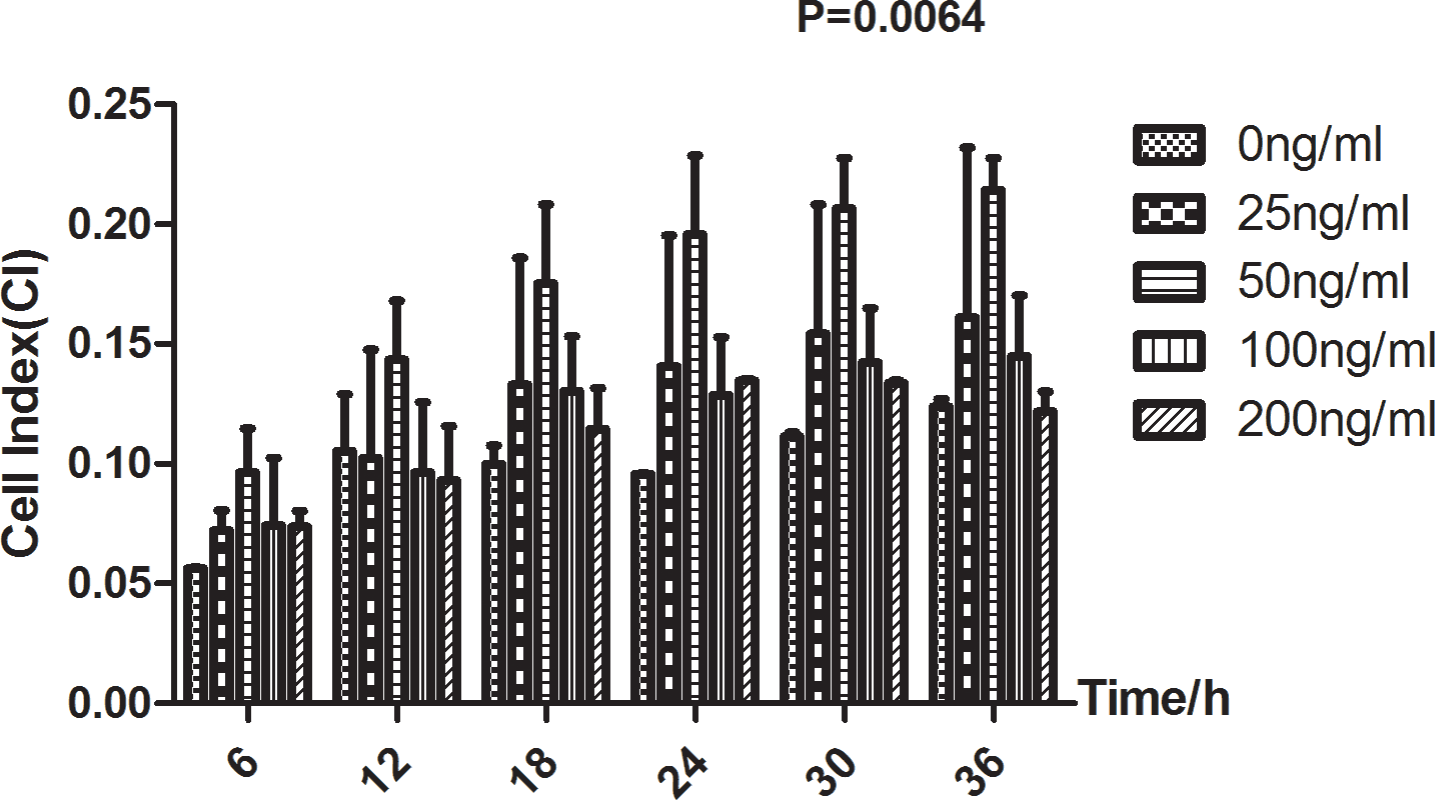
hFOB growth *in vitro* recorded at multiple time points under various Anxa2 protein concentrations (0-200ng/ml). Presented are cell indexes (CI) monitored for 36 hours in cell growth assay. The data is described as mean±SD. It reflected the inverse U-shaped effect of Anxa2 protein on hFOB growth at each representative time point (P = 0.0064).

In addition, **Fig 4** reflected the inverse U-shaped effect of Anxa2 more intuitively at the specific time points. At each set time point in **Fig 4**, low dose of Anxa2 promoted osteoblastic growth and the stimulatory effect peaked around the dose of 50ng/ml, and even higher dose (>50ng/ml) seemed to attenuate osteoblastic growth (P = 0.0064).

## Discussion

In this study, we firstly established the relevance of PBM-expressed Anxa2 protein to OF in humans. PBM-expressed Anxa2 was found significantly elevated in OF patients versus NF subjects. Referring to previous reports on significant up-regulation of PBM-expressed Anxa2 protein in low vs. high BMD subjects [10, 11], our observations point to the fact that Anxa2 protein sustains higher level of expression in PBM in subjects with poor bone (either low BMD or OF) than healthy controls. The evidences imply that Anxa2 probably play a crucial role during OP progression and might serve as a potential risk biomarker for OF.

This study represented the pioneering efforts in quantifying Anxa2 protein level in plasma and testing its association with hip BMD in humans. We found plasma Anxa2 level was negatively correlated with hip BMD in Chinese elderly, and significantly elevated in individuals with extremely low BMD versus high BMD. The association trend for Anxa2 and BMD, identified herein in plasma, is consistent with that reported in PBM [10, 11]. These observations coincide with and can be explained by the knowledge that Anxa2 could be secreted by both osteoclasts and monocytes, serving as an autocrine factor to stimulate monocytes trans-endothelial migration and/or osteoclastogenesis [10]. It was reported that Anxa2 expanded the osteoclasts precursors pool in human marrow cultures and facilitated osteoclasts formation [16]. Nesbitt and Horton proved that Anxa2 played an important role in the clearance of degraded bone matrix by osteoclasts [17]. These data supported that Anxa2 protein was a stimulator of osteoclast formation and activity [12].

However, the roles of Anxa2 in bone remodeling are not limited to the aspect of osteoclastogenesis. Functional experiments in mouse osteoblastic cells suggested that Anxa2 could promote mouse osteoprogenitor proliferation and differentiation, hence affect bone formation [13]. As observed in hFOB cell culture in the present study, Anxa2 could regulate human osteoblastic growth in a concentration-dependent manner. Specifically, lower dose (0-50ng/ml) of extracellular Anxa2 protein may significantly promote osteoblastic growth. However, even higher Anxa2 protein (>50ng/ml) could attenuate osteoblastic growth. Such trend of dose-response relationship also coincides with and explains the observed phenomenon of higher plasma Anxa2 level in low BMD subjects versus high BMD subjects in Sample 2.

Taken together, the present findings of significant up-regulation of Anxa2 in either low BMD or OF subjects are consistent with and partially supported by known functions of Anxa2 protein in osteoclastogenesis [13, 18]. Previous studies in genetically manipulated mice indicated that the activated osteoclast itself could be the source of an activity that contributes to the fine control of the coupling process [19]. Additional evidence supporting the idea that the coupling activity is generated from active osteoclasts came from Nakamura et al [20]. Consistently, a review on the communication between osteoclasts and osteoblasts suggested mature osteoclasts may interact not only with bone lining cells but also with osteoblasts to remove the bone collagen left by osteoclasts in resorption lacunae [21, 22]. What’s more, a recent study demonstrated that osteoclast-derived exosome miR-214-3p was secreted to inhibit osteoblasts activity *in vitro* and reduce bone formation *in vivo* simultaneously [23]. In combination, these studies implicate that the coupling process is precisely regulated and the osteoclasts could collaborate with other factors to influence the activity of osteoblast lineage. In light of the presence of Anxa2 in plasma and its association with hip BMD, its promotive roles in osteoclastogenesis, as well as its dual reverse roles in regulating osteoblastic growth, herein we propose a new concept as follows. Anxa2 protein, secreted into plasma by PBMs and/or osteoclasts, probably plays significant roles in coupling and tuning bone formation with bone resorption *in vivo*. The concentration-dependent dual effects of Anxa2 on osteoblast growth, as observed in this study, imply that the coupling process and the bone formation activity may be orchestrated by the resorption activity through an osteoclastic protein concentration-dependent manner. Anxa2 protein, secreted by PBMs/osteoclasts and present in plasma, may serve as a key molecule involved in osteoclast-osteoblast communication and bone remodeling.

## Conclusion

We concluded that PBM-expressed Anxa2 protein level is associated with hip OF and plasma Anxa2 protein level is negatively correlated with hip BMD in Chinese elderly. Furthermore, Anxa2 regulates osteoblastic growth in a concentration-dependent manner. Its promoting effect on osteoblastic growth could be attenuated at higher concentration (>50 ng/ml), which explains the observed phenomenon of higher plasma Anxa2 level in low BMD subjects. Besides its previously recognized role in promoting osteoclastogenesis, this study suggested that higher level of Anxa2 protein, which is expressed by PBM and secreted into plasma may attenuate osteoblastic growth, and be associated with hip BMD and OF in humans. Certainly, whether plasma Anxa2 protein level is predictive of OP/OF has yet to be investigated further by longitudinal studies.

## Supporting information

S1 Fig Scatter plot regarding the PBM-expressed Anxa2 protein level and the hospital entry day for the OF patients in Sample 1. Presented are Anxa2 protein abundances (Label-Free Quantification [LFQ] intensity) of 45 OF patients in PBM in Sample 1. The plot presents PBM-expressed Anxa2 protein level for 15 pooled samples from the 45 cases. The entry day was averaged on the 3 original cases for each pooled sample.
(TIF)

S2 Fig Standard curve for plasma Anxa2 assay in Sample 2. Four-parameter logistic fitting model was generated to test the fitting degree of standard curve by following the Anxa2 ELISA kit instructions (r = 0.99). X values represent concentrations of standards and Y values represent optical density (O.D.) values at 450nm.
(TIF)

S3 Fig Growth curve of hFOB cell cultures supplemented with human Anxa2 protein at various concentrations (0-200ng/ml). Presented are cell indexes (CI) recorded at every 10-minutes interval in real time for 36 hours.
(TIF)
