# Supplementary figures and images for: Anxa2 attenuates osteoblast growth and is associated with hip BMD and osteoporotic fracture in Chinese elderly
Source: PLoS One. 2018 Mar 23;13(3):e0194781. doi: 10.1371/journal.pone.0194781 (PMC5865729; doi:10.1371/journal.pone.0194781)

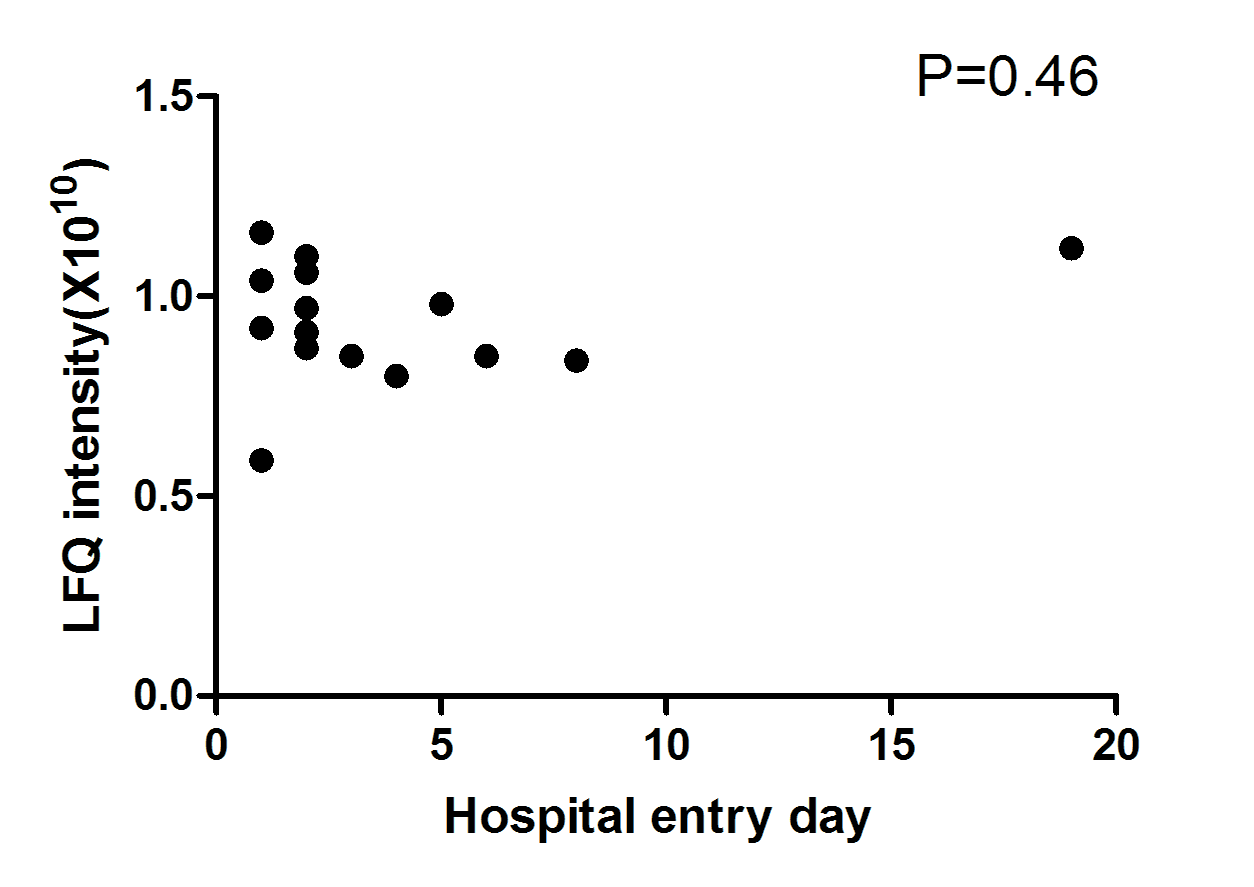

Supplement: S1 Fig — Presented are Anxa2 protein abundances (Label-Free Quantification [LFQ] intensity) of 45 OF patients in PBM in Sample 1. The plot presents PBM-expressed Anxa2 protein level for 15 pooled samples from the 45 cases. The entry day was averaged on the 3 original cases for each pooled sample. (TIF) [file pone.0194781.s001.tif]

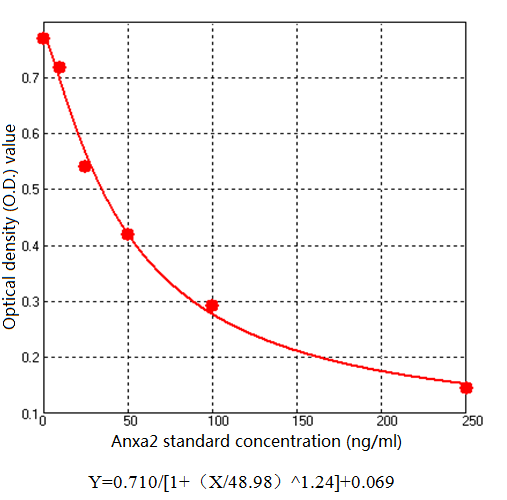

Supplement: S2 Fig — Four-parameter logistic fitting model was generated to test the fitting degree of standard curve by following the Anxa2 ELISA kit instructions (r = 0.99). X values represent concentrations of standards and Y values represent optical density (O.D.) values at 450nm. (TIF) [file pone.0194781.s002.tif]

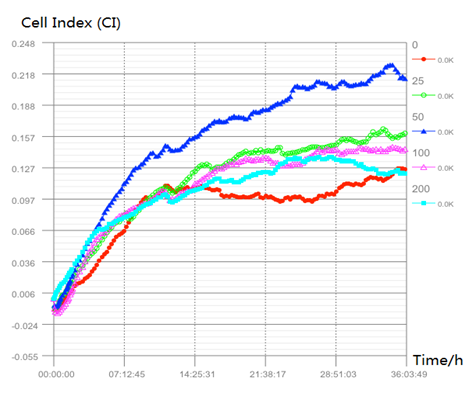

Supplement: S3 Fig — Presented are cell indexes (CI) recorded at every 10-minutes interval in real time for 36 hours. (TIF) [file pone.0194781.s003.tif]
